# Do we all perceive experiences of age discrimination in the same way? Cross-cultural differences in perceived age discrimination and its association with life satisfaction

**DOI:** 10.1007/s10433-023-00790-x

**Published:** 2023-11-16

**Authors:** M. Clara P. de Paula Couto, Jana Nikitin, Sylvie Graf, Helene H. Fung, Thomas M. Hess, Shyhnan Liou, Klaus Rothermund

**Affiliations:** 1 Institute of Psychology, Friedrich Schiller University Jena, Jena, Germany; 2 Department of Developmental and Educational Psychology, University of Vienna, Vienna, Austria; 3 Institute of Psychology, Czech Academy of Sciences, Brno, Czech Republic; 4 Department of Psychology, Chinese University of Hong Kong, Shatin, Hong Kong; 5 Department of Psychology, North Carolina State University, Raleigh, USA; 6 Institute of Creative Industries Design, National Cheng Kung University, Tainan, Taiwan

**Keywords:** Cultural differences, Ageism, Psychological well-being, Adaptation

## Abstract

Age discrimination is pervasive in most societies and bears far-reaching consequences for individuals’ psychological well-being. Despite that, studies that examine cross-cultural differences in age discrimination are still lacking. Likewise, whether the detrimental association between age discrimination and psychological well-being varies across contexts remains an open question. In this study, therefore, we examined cross-cultural differences in perceived experiences of age discrimination and their detrimental association with a specific indicator of psychological well-being, which is life satisfaction. The sample was drawn from the Ageing as Future study and comprised 1653 older adults (60–90 years) from the Czech Republic, Germany, Hong Kong, Taiwan, and the USA. Participants self-reported their experiences of age discrimination and their life satisfaction. Findings indicated that participants from Hong Kong and Taiwan reported experiences of perceived age discrimination more often than participants from the Czech Republic, Germany, and the USA. Furthermore, experiences of age discrimination were negatively associated with life satisfaction. Cultural context moderated this relation: We found a *smaller* detrimental association between perceived experiences of age discrimination and life satisfaction in Eastern cultures, that is, in contexts where such experiences were perceived to be *more* prevalent. These findings highlight the importance of examining age discrimination across cultures. Experiences of age discrimination are clearly undesirable in that they negatively affect psychological well-being. Our results indicate that a higher self-reported prevalence of perceived age discrimination in the samples studied weakens this negative association. We discuss these findings in terms of adaptation (versus sensitization) in response to discrimination.

**Supplementary Information:**

The online version contains supplementary material available at 10.1007/s10433-023-00790-x.

*Ageism* encompasses age-related negative thoughts, feelings, and actions, and it reflects stereotypes (i.e., negative beliefs), prejudice (i.e., negative evaluations), and discrimination against different age groups, including not only older (Butler 1969; Palmore 1999, 2001) but also younger adults (i.e., *youngism*, Bratt et al. 2018; Francioli and North 2021). *Age discrimination* is a label for situations in which people are excluded or disadvantaged due to their age. It includes practices and behaviors towards people that prevent them from participating in professional and social activities. This study aims to examine cross-cultural differences in perceived experiences of age discrimination reported by older adults. The primary goal of this study is however to investigate whether perceived experiences of age discrimination are differently associated with psychological well-being depending on specific contexts.

Age discrimination is quite prevalent in Western societies. For example, data collected in 29 European countries and Israel showed that approximately 43% of adults over 70 years had experienced age discrimination more than once in the past year (Vauclair et al. 2016). In addition to that, using data from 25 European countries, Rychtaříková (2019) reported that at least 24% of people (Denmark) thought that discrimination against people aged 55 or older was very or fairly widespread in their society. This proportion was highest in Bulgaria (63%) and the Czech Republic (60%). The situation is no different in the United States. Recent data showed that 45% of Americans aged 50–80 years have experienced ageism in their interpersonal interactions and that 65% reported exposure to ageist messages (Ober Allen et al. 2022).

Whereas the prevalence of age discrimination in European countries and in the United States has been extensively documented, much less is known about how frequently older persons in Eastern countries experience age discrimination. One study conducted in South Korea showed that 48% of participants aged 60 years and older in their sample had reported experiences of age discrimination (Kim et al. 2016). Although informative, the study by Kim and colleagues (2016) focused on a single Eastern culture. Hence, it remains an open question whether individuals from other Eastern cultural contexts experience age discrimination to the same extent as those individuals from Western cultures.

Likewise, even though there is plenty of evidence showing that age discrimination is not only pervasive but also harmful for mental health and psychological well-being (Ober Allen 2016; for reviews, see Chang et al. 2020; de Paula Couto and Rothermund 2019; Rothermund et al. 2021), it remains unclear whether such harmful effects are the same for everyone and across all situations. For example, previous studies in other research areas have shown that the extent to which negative experiences are shared in a context is associated with health-related outcomes (e.g., Heggebø and Elstad 2018). These findings indicate that context may indeed be a factor in determining to what degree experiences of age discrimination are harmful to people's physical and mental health (or other relevant outcomes). Therefore, in this study we are particularly interested in investigating the association between age discrimination and a specific indicator of psychological well-being, which is life satisfaction, depending on the prevalence of perceived experiences of age discrimination within different contexts.

Hence, with the current study we aim to fill in two gaps in the existing age discrimination literature. The first regards the examination of cross-cultural differences in perceived experiences of age discrimination across European, North American, and Eastern countries. The second relates to extending the current knowledge on the negative association between age discrimination and health and well-being by investigating whether this association is context dependent.

## Cross-cultural differences in perceived age discrimination

Regarding cross-cultural comparisons, many studies have been guided by the idea that the contrast between Eastern and Western cultures is an important proxy for cultural differences (for a recent review see Kornadt et al. 2022). Accordingly, it is assumed that attitudes towards older adults might be more positive in Eastern, collectivist countries than in Western, individualist countries due to the values that are emphasized in these cultural contexts. This *values hypothesis* is in line with the notion that collectivism emphasizes the importance of community, selflessness, interdependence, and generosity (Markus and Kitayama 1991), with a strong focus on Confucian values that prescribe, for example, that children must love, respect, and support their parents as well as other older adults in their family. Such a collectivist approach is thought to reduce intergenerational conflict because generations are assumed to be generous and loyal to each other, with older adults being held in high esteem (but see, e.g., Ayalon and Roy 2022). Based on the values hypothesis, we would thus expect that perceived age discrimination would be more often reported in Western than in Eastern cultural contexts.

Alternatively, age stereotypes have been claimed to be a precursor of perceived age discrimination (Voss et al. 2017a, b; Voss et al. 2018a), with negative age-related beliefs shaping behaviors towards older adults. In other words, negative age stereotypes would be associated with more perceptions of age discrimination. Thus, in line with this *age stereotypes hypothesis*, we would predict that perceived age discrimination is more often reported in contexts where age stereotypes are more negative. If, as shown by previous studies (de Paula Couto et al. 2022c; North & Fiske 2015; Voss et al. 2018b), age stereotypes are more negative in Eastern cultures, then we would predict that perceived age discrimination would be more often reported in Eastern than in Western cultures. We therefore test two cross-cultural hypotheses that are based on different theoretical perspectives and make competing predictions.

## Perceived age discrimination and life satisfaction

Age discrimination has been shown to negatively impact physical and mental health (Luo et al. 2012; Ober Allen et al. 2022; Shippee et al. 2019). The psychological consequences of age discrimination include lower levels of subjective well-being and self-esteem as well as lower levels of life satisfaction (for reviews, see Chang et al. 2020; Schmitt et al. 2014; see also Garstka et al. 2004). Experiences of age discrimination may be harmful because they can reveal the existence of exclusion, rejection, and negative treatment, which threatens the fulfillment of needs for inclusion and acceptance (Schmitt and Branscombe 2002; Schmitt et al. 2014). In addition to the negative effects of age discrimination resulting from interpersonal threats, age discrimination may also create structural barriers that reduce opportunities to access various resources, such as employment, health care, and income, and as a result, negatively affect well-being (Major et al. 2002; Schmitt et al. 2014).

The detrimental association between age discrimination and psychological outcomes is well-established in the literature, but whether the relationship between age discrimination and indicators of well-being varies across cultural contexts is less known. As mentioned above, contexts may differ in the reported prevalence of perceived age discrimination. Hence, one factor that might moderate the strength of this relationship is the prevalence of perceived age discrimination in a given context. Such moderating effect of an event’s prevalence on the psychological consequences of the respective event has already been investigated for other negative experiences: For instance, it was found that the detrimental effects of unemployment on well-being, health, and even mortality were *less severe* when the overall unemployment rate was *higher* (Heggebø and Elstad 2018; Martikainen and Valkonen 1996; Scanlan and Bundy 2009). A theoretical explanation for this pattern of results can be derived from an *adaptation hypothesis* that assumes the possibility that individuals *adapt* (or habituate) to frequent negative experiences, and in that case show *a reduced response* to such experiences (Frederick and Loewenstein 1999). Adaptation can occur as a result of one’s own repeated exposure to negative events but also by witnessing or visualizing others’ negative experiences, which can lead to *vicarious* habituation (Bandura et al. 1963; Swogger et al. 2014). In contexts where specific negative events are frequent, vicarious habituation can indirectly reduce the individual’s response to similar negative events. However, not all studies found support for adaptation to repeated stressful events, at least not in relation to experiences of unemployment (Luhmann and Eid 2009, see also Lucas et al. 2004). In these studies, *frequent* experiences of unemployment were associated with *lower* life satisfaction, reflecting a *sensitization* in response to such repeated exposure (i.e., a *sensitization hypothesis*).

Regarding cross-cultural comparisons of perceived experiences of age discrimination, it remains to be examined how individuals from different cultures respond to age discrimination, depending on how prevalent it is in each context. In our study, we use life satisfaction as an indicator of psychological well-being (Garstka et al. 2004; Schmitt et al. 2014). Drawing on research findings on unemployment, we investigate whether the negative association between age discrimination and life satisfaction depends on how frequently age discrimination is perceived in a given cultural context. We again test two competing hypotheses: according to a *sensitization hypothesis*, we predict that perceived age discrimination should be *more strongly* associated with life satisfaction in contexts where its prevalence is higher. In line with an *adaptation hypothesis* however, we would predict that age discrimination should be *less strongly* associated with life satisfaction in contexts where its prevalence is higher.

## The present study

In this study, we use data covering the age range from 60 to 90 years to examine cross-cultural differences in perceived experiences of age discrimination (PAD), considering three Western (the Czech Republic, Germany, and the United States) and two Eastern cultures (Hong Kong and Taiwan). Most importantly, we investigate the association between PAD and life satisfaction (LS), assuming that PAD is detrimental to LS. Additional tests focus on the moderating role of culture in the association between PAD and LS. Specifically, we want to test whether the strength of the association between PAD and LS differs between cultural contexts depending on the reported prevalence of PAD.

## Method

### Sample

Data were drawn from the third wave of the Ageing as Future questionnaire study (AAF, Lang et al. 2022), comprising participants from five different cultures that may have different experiences with ageism. In the current study, we used data from participants aged 60 years and older because the specific experiences of age discrimination we examined are most likely to be experienced by older adults. The final sample of the study consisted of 1,653 persons aged 60–90 years (53% female, *M*_age_ = 73.37, *SD* = 8.25), from the Czech Republic (*n* = 336), China (Hong Kong, *n* = 292, and Taiwan, *n* = 337), Germany (*n* = 457), and the United States (*n* = 231). Table 1 provides an overview of the sociodemographic information of the samples in the five examined countries.

**Table 1:** Sociodemographic Information by Culture

Variable	CZ (*n* = 336)	DE (*n* = 457)	HK (*n* = 292)	TW (*n* = 337)	USA (*n* = 231)
Age, *M (SD)*	74.0 (8.48)	73.9 (8.24)	72.6 (8.27)	73.4 (8.32)	72.5 (7.70)
Gender (% female)	55.7	50.5	54.1	55.2	50.6
Marital status (% married)	37.8	71.8	68.4	75.1	58.4
Health,* M (SD)*	2.10 (1.05)	2.39 (1.02)	2.25 (.78)	2.26 (.81)	3.13 (.81)
Education, *M (SD)*	4.43 (2.11)	4.97 (1.80)	2.52 (1.58)	2.31 (1.78)	6.02 (1.23)
Income^a^, *M (SD)*	2.32 (1.11)	4.83 (1.20)	3.76 (2.16)	2.62 (1.58)	5.90 (1.46)

CZ, Czech Republic; DE, Germany; HK, Hong Kong; TW, Taiwan; USA, United States

^a^Monthly household income after taxes was assessed on an 8-point scale ranging from 1 (0–500 Euro) to 8 (> 10,000 Euro)

To test cultural differences in PAD, we conducted chi-square tests and one-way analyses of variance (ANOVAs). Because of the heterogeneity of variances, we report results of the Welch test for the ANOVAs. The samples in each country were balanced regarding gender, *X*^*2*^(4) = 3.34, *p* = 0.502, and age, *F*(4, 767.40) = 2.12, *p* = 0.076, but differed regarding marital status (i.e., married vs. not married), *X*^*2*^(4) = 133.58, *p* < 0.001, education, *F*(4, 777.42) = 324.51, *p* < 0.001, monthly household income, *F*(4, 723.84) = 389.14, *p* < 0.001, and subjective health, *F*(4, 770.87) = 58.42, *p* < 0.001. Post hoc tests indicated that US-Americans had the highest level of education, followed by Germans and Czechs. Chinese (Hong Kong and Taiwan) had the lowest level of education. US-Americans also reported the highest monthly household income, followed by Germans and Hong Kong Chinese. Czechs and Taiwan Chinese reported the lowest monthly household income. Likewise, US-Americans reported better subjective health than all other countries in the sample. Regarding marital status, Taiwan and Hong Kong Chinese as well as Germans reported more often being married compared to US-Americans and Czechs (see Table 1).

### Procedures

Participants were randomly recruited via mail or telephone based on large databases that we received from local registry offices or from professional marketing companies. Even though participants were recruited through different means, we used the same stratified random sampling (balanced design) strategy in all countries such that the samples were stratified by age cohort and gender. The number of sampled participants in each stratum was set to *n* = 50 in all countries (see Lang et al. 2022 for a detailed description of the Methods).

After providing consent, participants answered the same self-administered questionnaire. In case participants had low literacy skills or vision problems, a trained interviewer was available for assistance. After completing and returning the questionnaire, participants received approximately a $20 compensation (either as a gift card or in cash). Research procedures were approved by the Institutional Review Boards at Friedrich-Schiller-University Jena, North Carolina State University, Chinese University of Hong Kong, University of Basel, and National Cheng Kung University.

### Measures

The measures used in the current study were part of a larger questionnaire developed in German and then translated (and back translated) into Chinese, Czech, and English for the respective surveys. The questionnaire included a wide range of variables that assessed different aspects of old age and ageing. In this study, we investigated self-reported perceived experiences of age discrimination and life satisfaction. For both measures, we report aggregated values across 11 items referring to different life domains (family and one’s committed relationships, friendships and acquaintances, independence and autonomy, leisure activities and commitment, personality and life management, finances and dealing with money, work and professional life, physical and mental fitness, appearance, and health).

#### Perceived experiences of age discrimination

We assessed perceived experiences of age discrimination (PAD) using a scale that we developed and tested in this study (see the Appendix A for the PAD scale items). We decided to develop a scale of perceived own experiences of age discrimination because previous studies that investigated perceived age discrimination focused either on overall experiences (e.g., Trusinová, 2014; Vauclair et al. 2015, 2016) or on the experiences made in only a few domains (e.g., Voss et al. 2017b). We did not find any domain-specific scales that included a wide range of life contexts, which was an important feature in the context of the AAF questionnaire study. For each item, participants indicated how often they have been discriminated due to their age in the 11 life domains listed above, on a 5-point scale ranging from 1 (“never”) to 5 (“very often”). Internal consistency of the scale was *α* = 0.93 and 0.94 in China (Hong Kong and Taiwan, respectively), 0.93 in the Czech Republic, 0.87 in Germany, and 0.85 in the United States.

#### Life satisfaction

We assessed life satisfaction with a scale developed and used in previous studies (see Kornadt and Rothermund 2011; Voss et al. 2017a). Participants rated their level of life satisfaction in the 11 life domains listed above, on a 5-point scale ranging from 1 (“very unsatisfied”) to 5 (“very satisfied”). Internal consistency of the scale was *α* = 0.90 and 0.94 in China (Hong Kong and Taiwan, respectively), 0.91 in the Czech Republic, 0.90 in Germany, and 0.88 in the United States.

#### Covariates

Covariates included were those sociodemographic variables that differed between cultures and/or correlated with perceived experiences of age discrimination (see Additional file 1: Table S1 in the Supplemental Materials). These were age, education level, subjective health, and marital status. We did not include monthly household income^1^ because the regression slopes across countries were not homogeneous (i.e., a moderation by income across the examined cultures). Education level was coded in accordance with the guidelines provided in the ISCED 2011 (1 = primary education, 2 = lower secondary education, 3 = upper secondary education, 4 = postsecondary non-tertiary education, 5 = short-cycle tertiary education, 6 = bachelor’s or equivalent level, 7 = master’s or equivalent level, 8 = doctoral or equivalent level; UNESCO Institute for Statistics 2012). Participants rated their subjective health status with a single item “How would you describe your current state of health?” with a 5-point scale ranging from 1 (“not good at all”) to 5 (“very good”).

### Statistical analyses

#### Measurement invariance

To address the potential issue regarding context specificity in the two assessed constructs (i.e., perceived age discrimination and life satisfaction) we carried out multiple-group confirmatory factor analyses (CFA) as well as measurement invariance analyses. We tested a single factor solution for PAD across the five included cultures. Due to missing values on items assessing PAD, 74 participants were not included in the analyses (*N* = 1579). Measurement invariance was tested in a stepwise manner (configural, metric, scalar). When it was not achieved, a close investigation of the modification indices allowed identification of the most non-invariant parameters in each step, which were then gradually released to assess partial invariance (for detailed information about the measurement invariance analyses, see the Supplemental Materials, Additional file 1: Table S2). The resulting model (partial scalar measurement invariance) with the five cultures had an acceptable fit, *X*^2^(282) = 557.441, CFI = 0.982, RMSEA = 0.051, and SRMR = 0.067. The change in the comparative fit index (CFI) and in the root-mean-square error of approximation (RMSEA) and standardized root-mean square residual (SRMR) were within the suggested guidelines (Chen 2007).

As with the PAD scale, we also tested a single factor solution for the life satisfaction scale across the five examined cultures. Due to missing values on items assessing life satisfaction, 110 participants were not included in the analyses (*N* = 1543). For detailed information about the measurement invariance analyses on life satisfaction, see the Supplemental Materials (Additional file 1: Table S3). The resulting model (partial scalar measurement invariance) with the five cultures had an acceptable fit, *X*^2^(288) = 769.148, CFI = 0.979, RMSEA = 0.069, and SRMR = 0.081. The change in the comparative fit index (CFI) and in the root-mean-square error of approximation (RMSEA) and standardized root-mean square residual (SRMR) were within the suggested guidelines (Chen 2007). These results as well as the results regarding the measurement invariance in the PAD scale indicate therefore the assessment of common constructs across the five investigated cultural contexts.

#### Analyses of covariance

To compare perceived experiences of age discrimination across the five countries, we conducted an ANCOVA. In this analysis, we included self-reported experienced ageism as the dependent variable and age, education level, marital status (dummy-coded), and subjective health as covariates.

#### Multiple regressions

To test our hypothesis that perceived experiences of age discrimination (PAD) are detrimental to life satisfaction, and to examine whether this association depends on cultural context (i.e., moderation by culture), we conducted a two-step hierarchical moderated multiple regression analysis. In this analysis, cultures were split into Eastern (Hong Kong and Taiwan) and Western (Czech Republic, Germany, and the United States).^2^ To control for any effects of the covariates, age, education level, marital status (dummy-coded), and subjective health were included in Model 1. The main effect of PAD and its interaction with culture were entered in Model 2 (variables were centered to allow simultaneous interpretation of main effects and interactions; Aiken and West 1991).

## Results

### Cross-cultural differences in perceived experiences of age discrimination

**Fig. 1:**
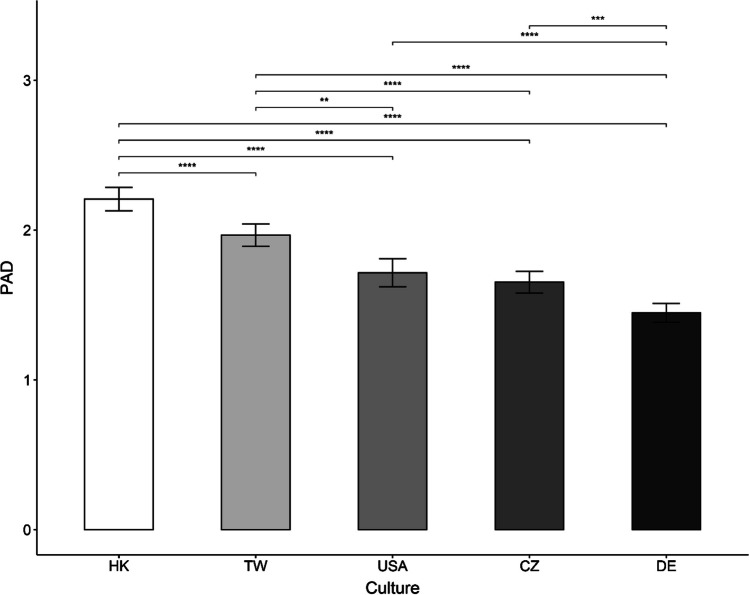
Pairwise Comparisons (Bonferroni Adjustment) for Adjusted Means of Self-Reported Perceived Experiences of Age Discrimination (PAD) Across Cultures. *Note.* PAD = Perceived experiences of age discrimination; CZ = Czech Republic; DE = Germany; HK = Hong Kong; TW = Taiwan; USA = United States. Whiskers indicate ± 1 Standard Error

Results of the ANCOVA across cultures with PAD as the dependent variable and age, education level, marital status, and subjective health as covariates showed a significant main effect of culture, *F*(4,1587) = 54.99, *p* < 0.001, *np*^2^ = 0.12. The main effect of subjective health was also significant, *F*(4,1587) = 35.81, *p* < 0.001, *np*^2^ = 0.05 (see Additional file 1: Table S4 in the Supplemental Materials for detailed results of the ANCOVA). Pairwise comparisons with Bonferroni adjustment indicated that perceived experiences of age discrimination were more often reported in Hong Kong (*M* = 2.22, *SE* = 0.041), followed by Taiwan (*M* = 1.98, *SE* = 0.040), the United Stated (*M* = 1.72, *SE* = 0.048) and Czech Republic (*M* = 1.65, *SE* = 0.037), and lastly by Germany (*M* = 1.46, *SE* = 0.033). Figure 1 depicts the adjusted means of PAD across the five examined cultures and their pairwise comparisons. Additional file 1: Table S5 in the Supplemental Materials shows the adjusted and unadjusted means and standard errors of PAD across the five cultures.

### Perceived experiences of age discrimination and life satisfaction

**Fig. 2:**
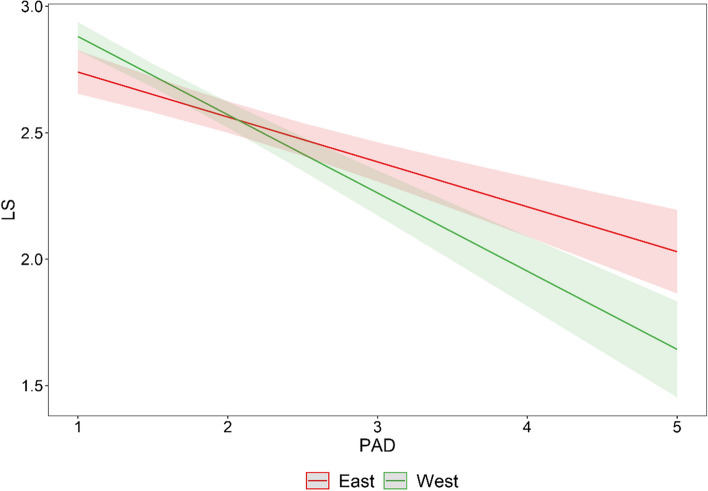
Interaction between Perceived Experiences of Age Discrimination and Culture (East x West) in Predicting Life Satisfaction. *Note.* PAD = Perceived experiences of age discrimination; LS = Life satisfaction

The results of the hierarchical regression analysis with life satisfaction (LS) as the dependent variable predicted by perceived experiences of age discrimination (PAD), culture, and their interaction are shown in Table 2. Model 2, which includes the main effect of PAD, culture, and their interaction showed a significant negative association between PAD and LS, *β* = − 0.20, *p* < 0.001. Most importantly, this main effect was qualified by culture, *β* = − 0.10, *p* < 0.001, with a stronger detrimental association of PAD and LS in Western, *β* = − 0.31, *p* < 0.001, than in Eastern, *β* = − 0.18, *p* < 0.001, cultural contexts (see Fig. 2).

**Table 2:** Hierarchical Regression Analysis of Predictors of Life Satisfaction

Predictor Variables	Model 1	Model 2
Age	.00	.00
Education level	.11***	.04
Marital status (married)	.05*	.05*
Perceived health	.50***	.43***
PAD		−.20***
Culture (Western cultural contexts)		.03
PAD × Culture		−.10***
*R*^*2*^	.293	.366
Δ*R*^*2*^	.293	.073
Δ*F*	163.57	60.83***

Standardized regression weights are presented. Culture is dummy coded; Eastern cultures are represented by Hong Kong and Taiwan, is the reference category. Western cultures are represented by the Czech Republic, Germany, and the United States. Marital status is dummy coded; not married is the reference category. PAD: Personal experiences of age discrimination. All predictors are centered. *p < .05;  **p < .01;  ***p < .001

## Discussion

The main findings of the present study are that (1) participants from different cultural contexts self-report different levels of perceived experiences of age discrimination, with participants from the Eastern cultures in our sample reporting more frequent perceived experiences of age discrimination than participants from the Western cultures in our sample; (2) perceived experiences of age discrimination are detrimental for life satisfaction; and (3) cultural context moderates this relationship, with perceived experiences of age discrimination being more strongly associated with life satisfaction in the assessed Western cultures, that is, in contexts where perceived experiences of age discrimination were reported as being *less* frequent.

### Cross-cultural differences in perceived experiences of age discrimination

Our findings that more age discrimination is reported in the assessed Eastern than in the assessed Western cultures are inconsistent with the values hypothesis that attitudes towards older people and old age are more positive in collectivist cultural contexts than in individualist ones (e.g., Ackerman and Chopik 2021). Instead, the pattern of cross-cultural differences in PAD identified in our study is in line with an age stereotypes hypothesis and extends previous research findings on differences in views on ageing in Eastern and Western regions (North and Fiske 2015; see also Vauclair et al. 2017; Voss et al. 2018a).

We acknowledge that comparing a small number of countries may fail to reflect cultural differences, however we believe that research undertaken in a small number of countries can still provide relevant insights into cultural aspects that are not yet well understood. The current knowledge of how perceived age discrimination varies across cultural contexts is only emerging. Hence, empirical evidence regarding the perceived prevalence of experiences of age discrimination across a small number of countries is an important first step in this respect. Our study included two Asian, one North American, and two European cultures. This was an initial effort to broaden the range of the cultural contexts studied so far, which were primarily European. That said, we do not intend to generalize from our sample of countries but to offer evidence that can be useful to support future cross-cultural work on age discrimination.

As previous research on age discrimination in the European region has shown, it is important to consider country-level variables. For example, Vauclair and colleagues (2015) demonstrated that the social status of older adults was perceived to be higher in countries with higher modernization rates (e.g., life expectancy, income, education, and urbanization) and with a higher proportion of employed older adults. Likewise, the role of meta-perceptions (i.e., older adults’ perception of how others in their social context perceive them) was shown to be an important predictor of age discrimination (Vauclair et al. 2016). The limited number of countries involved in our study did not allow us to include country-related variables as level-2 predictors to identify the sources of the observed differences in age discrimination. Regarding age discrimination, two interesting country-level indicators of differences in ageing contexts could be examined: (1) the Global AgeWatch Index (AgeInternational 2015) and (2) the relative age index (Ayalon and Rothermund 2018). These indicators have been shown to be associated with worse ageing conditions within countries. To sum up, the inclusion of more countries and/or additional variables will be important to better understand underlying cultural and environmental factors that contribute to experiences of age discrimination in specific contexts (Kornadt et al. 2022).

### Subjective versus objective age discrimination

Experiences of age discrimination depend on attributions of mistreatment or disadvantages to one’s age, which distinguishes these experiences from other types of discrimination (e.g., Voss et al. 2018a). In some cases the sources of mistreatment are obvious or are explicitly stated (e.g., when one is told to be too old for a job or an activity). We must concede, however, that in some cases, attributions to age are more speculative and might even be wrong in that the actual underlying reason for mistreatment in a certain situation are misperceived. Similarly, older people may attribute experiences of exclusion, rejection, or disadvantage to other causes (e.g., disability, obesity) although the actual cause of these incidents was their age. Self-reported age discrimination is thus not an objective measure of age discrimination and can either over-or underestimate actual discrimination because of one’s age. Furthermore, because people may occupy different disadvantaged statuses, measures that focus on a single status (e.g., age) may ignore that these different statuses may interact in shaping one’s experiences of discrimination. In line with that, theories of intersectionality question the idea that marginalized individuals perceive a single reason for their experiences of discrimination (Harnois et al. 2022).

Based on these considerations, we must acknowledge that our measure of age discrimination may provide a biased estimate of actual age discrimination. This also transfers to the interpretation of country differences, the estimation of which may also be biased by differences in the tendency to attribute negative experiences to one’s age between countries, rather than reflecting objective differences in age discrimination. We developed our measure of perceived age discrimination to be comprehensive and include a broad range of situations of discrimination that have been typically attributed to age in previous studies and in existing measures. Furthermore, our scale has shown good psychometric properties also showing measurement equivalence across all assessed samples. Besides, we used a one-stage approach in our measure (see e.g., Lewis et al. 2015, for a review), explicitly inquiring participants about experiences of discrimination due to their age in the beginning of the scale. Whether a two-stage approach (i.e., respondents are first asked about how their experiences of various forms of mistreatment and then asked to indicate the main reasons for these experiences) would reveal different patterns of age discrimination could be investigated in future studies.

### Perceived experiences of age discrimination and life satisfaction

Our findings regarding the PAD-LS association replicate previous studies showing that experiencing age discrimination negatively impacts mental health and well-being (e.g., Garstka et al. 2004; Ober Allen et al. 2022; Shippee et al. 2019). In our study, PAD was negatively associated with LS in all examined cultures. Interestingly, however, we also found a moderation of this relationship by culture, suggesting that in contexts where PAD is reported as less prevalent (as indicated by the cultural differences in this study), its negative association with life satisfaction is stronger. This pattern of results is in line with an adaptation hypothesis (Luhmann and Eid 2009; Lucas et al. 2004), which implies that frequently observing age discrimination in others may lead to vicarious habituation to these negative experiences or may be associated with the development of coping strategies that enable individuals to better handle adversities such as age discrimination. This form of adapting to adverse events contrasts with sensitization (i.e., a stronger response to negative events after repeated exposure), which would be reflected in the opposite pattern of results (i.e., a stronger detrimental association between PAD and LS when PAD occurs more frequently).

Another background factor that might contribute to the emergence of cultural differences in the consequences of PAD is the quality and prevalence of existing prescriptive age norms. In contexts where social expectations for older adults are dominated by strict norms that older adults should not be a burden and should altruistically disengage from social roles in favor of younger generations (de Paula Couto et al. 2022a; see also North and Fiske 2013a), age discrimination may be perceived as a social response to the violation of these norms (Gelfand et al. 2011). Accordingly, we did find higher levels of disengagement norms in Eastern compared to Western cultures (de Paula Couto et al. 2022b). Feelings of guilt or shame may arise from not living in accordance with these norms. Although speculative, we consider differences in prescriptive age norms to be a plausible source of differences in the extent of age discrimination and its consequences: Strict norms will shape behavior and thus reduce the incidence of discrimination against older people due to violations of these norms. At the same time, internalizing these norms may foster the negative effects of age discrimination among older people, who may tend to blame themselves––rather than others––for these experiences.

### Limitations and directions for future research

We have investigated five different cultural contexts that represent a selection of countries within Western and Eastern regions. In addition, even though our sample is stratified by age and sex, the sample sizes in each context are too small to be nationally representative. Hence, the data cannot be used to support claims about differences between national settings. The inclusion of representative samples, of other countries as well as the assessment of country-level variables (e.g., GDP, cultural values) would be an important step for future research that aims to examine cross-national differences in the experience of age discrimination. Moreover, as with cultural values, we have not included assessment of age-stereotypes in our study, which could have helped better explain and disentangle the values from the age-stereotypes hypotheses. However, we derived theses specific hypotheses from and interpreted our findings in line with previous empirical research that examined values and age stereotypes as precursors of age discrimination (Ackermann and Chopik 2021; North and Fiske 2015; Voss et al. 2018a).

In addition, interpretations of our findings are limited by the cross-sectional design of our study. Accordingly, relations between perceived age discrimination and life satisfaction should not be interpreted causally, as life satisfaction might also influence perceptions of age discrimination. Nonetheless, we believe that the inclusion of cultures representing Western and Eastern contexts and our large overall sample represent significant and innovative contributions to the literature.

Even though our study focused on cross-cultural differences in overall levels of age discrimination and its negative association with life satisfaction, we should point out that age differences and domain specificity of age discrimination are important topics for future research. Most of the reported evidence on age discrimination focuses on older adults’ experiences of discrimination in general (cf. e.g., Chasteen et al. 2021). However, researchers have pointed out that different age groups may be target of age-specific experiences of discrimination by being differently exposed to certain life contexts (North and Fiske 2013b). For example, very old adults may be more likely to face age discrimination in life domains that are related to shared resources, like the health care or the pensions systems. Differently, middle-aged adults may more easily become target of age discrimination in life domains related to “making way to the younger generations”, like the work context, for example. In light of this, future studies should also focus on the discrimination experiences of middle-aged and younger adults (Bratt et al. 2018). Moreover, previous studies emphasized the need to acknowledge that ageism can differ across behaviors and life domains. It may take different forms (e.g., paternalistic or hostile behaviors) and be more or less common in some life domains than in others (Voss et al. 2017a, b). Finally, examining the *severity* of experiences of age discrimination may represent an important step in understanding its harmful effects on psychological well-being indicators (Schmitt and Branscombe 2002).

## Conclusion

Age discrimination is a widespread phenomenon that affects people of almost all ages. Our findings of cross-cultural differences in perceived experiences of age discrimination highlight the contextual dependence of PAD. We hope that our study will stimulate further research on the contexts in which age discrimination occurs and the process by which it develops. Even though these contexts are diverse, it is not uncommon that all too often our scholarship seems to reflect only a small portion of this diversity.

Moreover, our findings showed that age discrimination affects life satisfaction regardless of its perceived prevalence and cultural context. However, the prevalence of PAD seems to play an important role in this association as adaptation in response to PAD occurs in contexts where PAD is perceived more frequently. Examining and identifying the underlying sources of these differences will be an important next step in improving our understanding of age discrimination and its consequences.

### Electronic supplementary material

Below is the link to the electronic supplementary material.

**Additional file 1.**

## Appendix A

Think about yourself; how often have *you* been discriminated against *due to your age* in different life domains?

**Table:**

I have been discriminated against in the domain of…	Never	Very rarely	Rarely	Often	Very often
(a) **… family and relationships** (e.g., I have been ignored or not taken seriously, I have been patronized)	□	□	□	□	□
(b) **… friendships and acquaintances** (e.g., I have been told disrespectful jokes about getting older, I have been judged for having younger friends)	□	□	□	□	□
(c) **… independence and autonomy** (e.g., I have been told to move to a residential home due to my age)	□	□	□	□	□
(d) **… leisure activities and volunteer commitments** (e.g., I have been told I am too old to do things, I have been excluded from activities)	□	□	□	□	□
(e) **… personality and life management** (e.g., I have been accused of being intolerant, rigid, stubborn, not open to novelty due to my age)	□	□	□	□	□
(f) **… finances and dealing with money** (e.g., I have had a loan refused, I have been told that I am no longer capable of managing my finances)	□	□	□	□	□
(g) **… work and professional life** (e.g., I have been denied a job, I have been disadvantaged at the workplace, I have been unable to continue working after the retirement age)	□	□	□	□	□
(h) **… physical fitness** (e.g., I have been helped without wanting it, I have been excluded from activities that demand physical effort)	□	□	□	□	□
(i) **… mental fitness** (e.g., I have had people speaking slowly and simply to me, I have had people making decisions for me)	□	□	□	□	□
(j) **… appearance** (e.g., I am pressured to look young)	□	□	□	□	□
(k) **… health** (e.g., I have been denied a specific medical treatment, I have had doctors dismissing symptoms because they attribute them to my age)	□	□	□	□	□

## Footnotes

1 The variable income ranges from 1 (0–500 Euro) to 8 (> 10,000 Euro) and was used in the following way: To make it comparable across the examined countries, the amount in Euro was converted in the local currencies according to exchange rates at the time we collected the data (so each category in the scale represents the same absolute value but in different currencies).

2 For the follow-up regression analysis, we grouped the cultures in our sample into Eastern and Western because the pattern of results regarding cross-cultural differences in PAD across the five cultures supports grouping them in these two broad cultural contexts, which also simplifies the follow-up analysis.
