# Supplementary material for: Do we all perceive experiences of age discrimination in the same way? Cross-cultural differences in perceived age discrimination and its association with life satisfaction
Source: Eur J Ageing. 2023 Nov 16;20(1):43. doi: 10.1007/s10433-023-00790-x (PMC10654333; doi:10.1007/s10433-023-00790-x)
Supplement: Supplementary file 1 — Additional file 1. [file 10433_2023_790_MOESM1_ESM.docx]

**Supplemental Materials**

| **Table S1**  *Correlations Between the Study Variables (N=1,653)* | | | | | | |
| --- | --- | --- | --- | --- | --- | --- |
|  | PAD | LS | Age | Education | Subj. Health | Income |
| PAD | 1 |  |  |  |  |  |
| LS | -.42** | 1 |  |  |  |  |
| Age | .07^**^ | -.13^**^ | 1 |  |  |  |
| Education | -.26^**^ | .23^**^ | -.12^**^ | 1 |  |  |
| Subj. Health | -.26^**^ | .52^**^ | -.23^**^ | .24^**^ | 1 |  |
| Income | -.19^**^ | .27^**^ | -.15^**^ | .42^**^ | .29^**^ | 1 |
| ***p*<.001. PAD=Perceived Experiences of Age Discrimination; LS=Life Satisfaction; Subj. Health=Subjective Health | | | | | | |

| **Table S2** | | | | |
| --- | --- | --- | --- | --- |
| *Perceived Experiences of Age Discrimination: Multigroup CFA results. Global fit measures for the exact measurement equivalence of the 11-item model, Countries: Czech Republic, Germany, Hong Kong, Taiwan, and the United States* | | | | |
|  | Chi2(df) | RMSEA | CFI | SRMR |
| configural | 344.604 (210)*** | 0.028 | 0.996 | 0.038 |
| metric | 407.280 (250)*** | 0.041 | 0.990 | 0.059 |
| scalar | 674.871 (290)*** | 0.060 | 0.975 | 0.073 |
| partial scalar | 557.441 (282)*** | 0.051 | 0.982 | 0.067 |
| *Note.* CFA=confirmatory factor analysis; RMSEA= robust root-mean-square error of approximation; CFI= robust comparative fit index; *SRMR= standardized root-mean-square residual. *** p<*0.001 | | | | |
|  |  |  |  |  |

| **Table S3** | | | | |
| --- | --- | --- | --- | --- |
| *Life Satisfaction: Multigroup CFA results. Global fit measures for the exact measurement equivalence of the 11-item model, Countries: Czech Republic, Germany, Hong Kong, Taiwan, and the United States* | | | | |
|  | Chi2(df) | RMSEA | CFI | SRMR |
| configural | 683.211 (220)*** | 0.055 | 0.997 | 0.043 |
| metric | 665.764 (260)*** | 0.071 | 0.979 | 0.086 |
| partial metric | 555.785 (248)*** | 0.059 | 0.990 | 0.072 |
| partial scalar | 769.148 (288)*** | 0.069 | 0.979 | 0.081 |
| *Note.* CFA=confirmatory factor analysis; RMSEA= root-mean-square error of approximation; CFI= comparative fit index; *SRMR= standardized root-mean-square residual. *** p <0.001; ** p <0.01; * p < 0.05* | | | | |
|  |  |  |  |  |

| **Table S4**  *Analysis of Covariance for Perceived Experiences of Age Discrimination (PAD) by Culture with Age, Education, Subjective Health, and Marital Status as Covariates* | | | | | | |
| --- | --- | --- | --- | --- | --- | --- |
| **Source** | **SS** | **df** | **MS** | **F** | **p** | **η_p_^2^** |
| Age | 0.52 | 1.00 | 0.52 | 1.28 | 0.26 | 0,00 |
| Education | 0.09 | 1.00 | 0.09 | 0.21 | 0.65 | 0,00 |
| Subjective Health | 35.81 | 1.00 | 35.81 | 87.69 | <.001 | 0.05 |
| Marital Status | 0.74 | 1.00 | 0.74 | 1.81 | 0.18 | 0.00 |
| Culture | 89.83 | 4.00 | 22.46 | 54.99 | <.001 | 0.12 |
| Error | 648.12 | 1587.00 | 0.41 |  |  |  |
| R Squared = .220 (Adjusted R Squared = .217) | | | | | | |
| *Note.* SS = sum of squares; MS = mean squares; effect size = η_p_^2^ | | | | | | |

| **Table S5**  *Unadjusted and Covariates Adjusted Descriptive Statistics for Perceived Experiences of Age Discrimination (PAD) Across Cultures* | | | | |
| --- | --- | --- | --- | --- |
| **Culture** | **PAD (Unadjusted)** | | **PAD (Adjusted)** | |
|  | **Mean** | **SE** | **Mean** | **SE** |
| CZ | 1.70 | .040 | 1.65 | .037 |
| DE | 1.44 | .024 | 1.46 | .033 |
| HK | 2.24 | .046 | 2.22 | .041 |
| TW | 2.00 | .038 | 1.98 | .040 |
| USA | 1.57 | .036 | 1.72 | .048 |
| *Note.* CZ = Czech Republic; DE = Germany; HK = Hong Kong; TW = Taiwan; USA = United States. | | | | |
